# One-year clinical evaluation of rotationally asymmetric multifocal intraocular lens with +1.5 diopters near addition

**DOI:** 10.1038/s41598-019-49524-z

**Published:** 2019-09-11

**Authors:** Tetsuro Oshika, Hiroyuki Arai, Yoshifumi Fujita, Mikio Inamura, Yasushi Inoue, Toru Noda, Kazunori Miyata

**Affiliations:** 1 Department of Ophthalmology, Faculty of Medicine, University of Tsukuba, Ibaraki, Japan; 2 Queen’s Eye Clinic, Kanagawa, Japan; 3 Fujita Eye Clinic, Tokushima, Japan; 4 Inamura Eye Clinic, Kanagawa, Japan; 5 Inoue Eye Clinic, Okayama, Japan; 6 Department of Ophthalmology, National Hospital Organization, Tokyo Medical Center, Tokyo, Japan; 7 Miyata Eye Hospital, Miyazaki, Japan

**Keywords:** Lens diseases, Reconstruction

## Abstract

We conducted a one-year prospective, multicenter study to assess clinical outcomes after implantation of segmented, rotationally asymmetric multifocal intraocular lenses (IOLs) with +1.5 diopters (D) near addition. In this phase III clinical trial, 120 eyes of 65 patients undergoing phacoemulsification and implantation of Lentis Comfort LS-313 MF15 (Oculentis GmbH) were included. The ophthalmological examinations were performed before and 1 day, 1 week, 1, 3, 6, 9, and 12 months after surgery. Uncorrected (UDVA) and corrected (CDVA) distance visual acuity, uncorrected (UIVA) and distance-corrected (DCIVA) intermediate visual acuity at 70 cm, and uncorrected (UNVA) and distance-corrected (DCNVA) near visual acuity at 30 cm were measured. A defocus curve was obtained and patients were asked about the severity of photic phenomena. Postoperative distance and intermediate visual acuity was excellent, with UDVA, CDVA, UIVA, and DCIVA of approximately 20/20, 20/16, 20/25, 20/25 were attained, respectively. The level of near visual acuity was lower; UNVA and DCNVA remained at around 20/60 and 20/70, respectively. The defocus curve indicated that postoperative uncorrected visual acuity of 20/25 and 20/40 was obtained at as close as 67 cm and 50 cm, respectively. Contrast sensitivity was within the normal range, with a minimal level of subjective symptoms and high patient satisfaction. The rotationally asymmetric multifocal IOLs with +1.5 D near addition provided excellent distance and intermediate vision, but near vision was not enough for reading small prints. Contrast sensitivity was high, with very low incidences of photic phenomena and a high level of patient satisfaction.

## Introduction

Multifocal intraocular lens (IOL) is designed to reduce patients’ spectacle dependence after cataract surgery. Conventional multifocal IOLs with two distinct foci can provide patients with good distance and near vision, but intermediate vision is not satisfactory^1–4^. These IOLs share the disadvantage of offering sharp vision only within a limited zone around the foci. In addition, the main drawbacks of conventional multifocal IOLs is the incidence and entity of reported side effects, including dysphotopsia and loss of contrast sensitivity^1–4^. There has been a growing demand in intermediate distance activities in everyday life, especially due to the more frequent use of personal computers and smartphones, leading to increased desire for spectacle independence in the intermediate range.

One trend that is surfacing today is the use of low-addition multifocal IOLs to boost intermediate performance and provide patients with good contrast sensitivity. One such IOL is the Lentis Comfort LS-313 MF15 (Oculentis GmbH, Berlin, Germany), a rotationally asymmetric multifocal IOL with +1.5 diopters (D) near addition. It is a plate-haptic IOL with a refractive segmented multifocal optic. Its concept is to provide patients with enhanced vision at a distance of 60 cm and more. There have been only a few studies which evaluated the clinical performance of this IOL^5–9^. In general, studies reported good visual performance in distance and intermediate distances, in combination with low incidence of subjective disturbing photic phenomena. In these studies, however, the number of eyes evaluated were small, 21 to 60 eyes, and follow-up period was short, 3 to 6 months, except for one study which assessed 21 patients for 12 months^8^. We conducted the current prospective, multicenter study to assess the clinical performance of plate-haptic, rotationally asymmetric multifocal IOLs with +1.5 D near addition in a larger cohort for 1 year.

## Patients and Methods

### Patient selection

The current prospective multicenter study was a phase III clinical trial of 12-month duration to evaluate Lentis Comfort LS-313 MF15 and to file for approval from the Ministry of Health, Labour and Welfare of Japan. The study included 120 eyes of 65 patients who were undergoing cataract surgery with IOL implantation. Their age was 41 to 88 years (69.7 ± 7.9 years, mean ± standard deviation), and there were 17 males and 48 females. They were selected from consecutive cases among the clinic population who matched our inclusion criteria. None of the eyes had any history of previous ocular surgery. Eyes were not included if they had any ocular diseases which can affect surgical outcomes. Eyes having corneal astigmatism greater than 1.5 D were also excluded from the study.

The institutional review boards at all surgical sites (Queen’s Eye Clinic, Fujita Eye Clinic, Inamura Eye Clinic, Inoue Eye Clinic, National Hospital Organization Tokyo Medical Center, and Miyata Eye Hospital) approved the study protocol, and a written informed consent was obtained from each patient before the study started. The study adhered to the tenets of the Declaration of Helsinki and good clinical practice for a medical device in Japan (Pharmaceuticals and Medical Devices Agency: PMDA clinical trial identifier: TC2). This study was registered at ClinicalTrials.gov ID: NCT02888210, https://clinicaltrials.gov/show/NCT02888210 (September 2, 2016).

### Intraocular lenses and surgery

All patients were submitted to the implantation of the same type of IOL. The specific IOL used for this study is a foldable plate-haptic hydrophilic acrylic IOL with hydrophobic surface properties, rotationally asymmetric, refractive multifocal IOL, combining an aspheric distance vision zone and a sector-shaped near vision zone with an add power of +1.5 D on the lens plane (Lentis Comfort LS-313 MF15, City and Country). According to the manufacture’s calculation, +1.5 D addition on the IOL planes translates to +1.06 D addition on the corneal plane. It has a 6.0-mm biconvex optic and an overall length of 11.0 mm^10–12^. All eyes were targeted emmetropia except for a few cases.

Six surgeons from 6 surgical sites performed surgeries. All surgeries were performed using a standard technique of sutureless phacoemulsification through a 2.3- or 2.4-mm incision. Anterior capsulorhexis of approximately 5.0 mm in diameter was created and the IOL was implanted into the capsular bag using a specific injector suggested by the manufacturer (Viscojet-BIO 2.2 injector. Medicel AG, Wolfhalden, Altenrhein or ACCUJECT UNIFIT WJ-60M II, Santen Pharmaceutical Co., Ltd. Osaka, Japan).

### Examinations

The ophthalmological examinations were performed before and 1 day, 1 week, 1, 3, 6, 9, and 12 months after surgery. Preoperative examination included measurements of uncorrected (UDVA) and corrected (CDVA) distance visual acuity, uncorrected (UIVA) and distance-corrected (DCIVA) intermediate visual acuity measured at 70 cm, uncorrected (UNVA) and distance-corrected (DCNVA) near visual acuity measured at 30 cm, manifest refraction, intraocular pressure, slitlamp anterior segment examination, optical biometry, keratometry, and retina evaluation under pupil dilation.

Postoperatively, UDVA, CDVA, UIVA, DCIVA, UNVA, and DCNVA were evaluated at all postoperative visits. A defocus curve for 15 different levels of defocus from +2.0 to −5.0 D in steps of 0.5 D was obtained at 6 months postoperatively. The contrast sensitivity was assessed at 3, 6, 12, and 18 cycles per degree using a CSV-1000 chart (Vector Vision, Greenville, OH) at 1, 3, 6, 9, 12 months postoperatively. The background illumination of the translucent chart was provided by the fluorescent luminance source of the instrument and was automatically calibrated to 85 cd/m^2^. To plot the curve, the measurement results were converted to log units using a specific table for the CSV-1000. Corneal endothelial cell count was assessed before and 6, 9, and 12 months after surgery. Endothelial cell density was evaluated using an automated noncontact specular microscope (SP-3000P, Topcon, Tokyo, Japan, or EM-3000, TOMEY, Aichi, Japan) by analyzing at least 50 cells of the central cornea. Three measurements were repeated and an image of the best quality was used for the analysis. Before starting this study, several meetings were held to standardize the surgical and measurement procedures as much as possible, and at least one technician from each site was trained for conducting reproducible examinations.

Patients were asked about the severity of photic phenomena. The intensity of glare and halo was graded as none, mild, moderate, or severe. The severity of difficulty in night vision was graded as none, mild to moderate, or severe. Patients were also asked about their overall satisfaction with the outcomes as follows, very high, high, medium, and low. The occurrence of any intraoperative and postoperative complications were recorded throughout the study period.

### Sample size calculation

Judging from the previous report that UIVA at 80 cm in eyes with Lentis-Mplus LS-312 MF15 IOL was 0.19 ± 0.11 logMAR^13^, the mean DCIVA at 70 cm in this study was estimated to be 0.17 logMAR. In order that the lower endpoint of 95% confidence interval of DCIVA in our study significantly exceeds the mean DCIVA in eyes with conventional monofocal IOL (0.22 logMAR), the required number of cases was calculated to be 77 eyes (α = 0.05, power 80%). In addition, ISO 11979-7:2014 (E) Annex B indicated that 100 eyes are required to statistically compare the rate of eyes with CDVA of 20/40 (0.3 logMAR) or better with the SPE rate (92.5%). A dropout rate of 10% was anticipated, giving the target sample size of 110 eyes.

## Results

**Figure 1:**
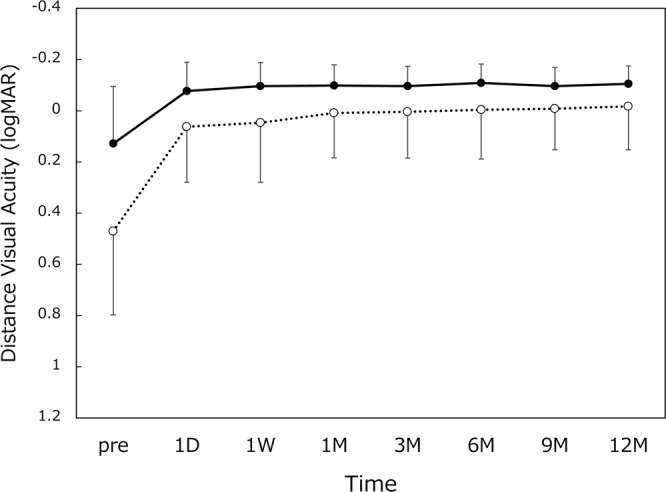
Uncorrected (UDVA, dotted line) and corrected (CDVA, solid line) distance visual acuity. logMAR = logarithm of minimum angle of resolution, mean ± standard deviation.

**Figure 2:**
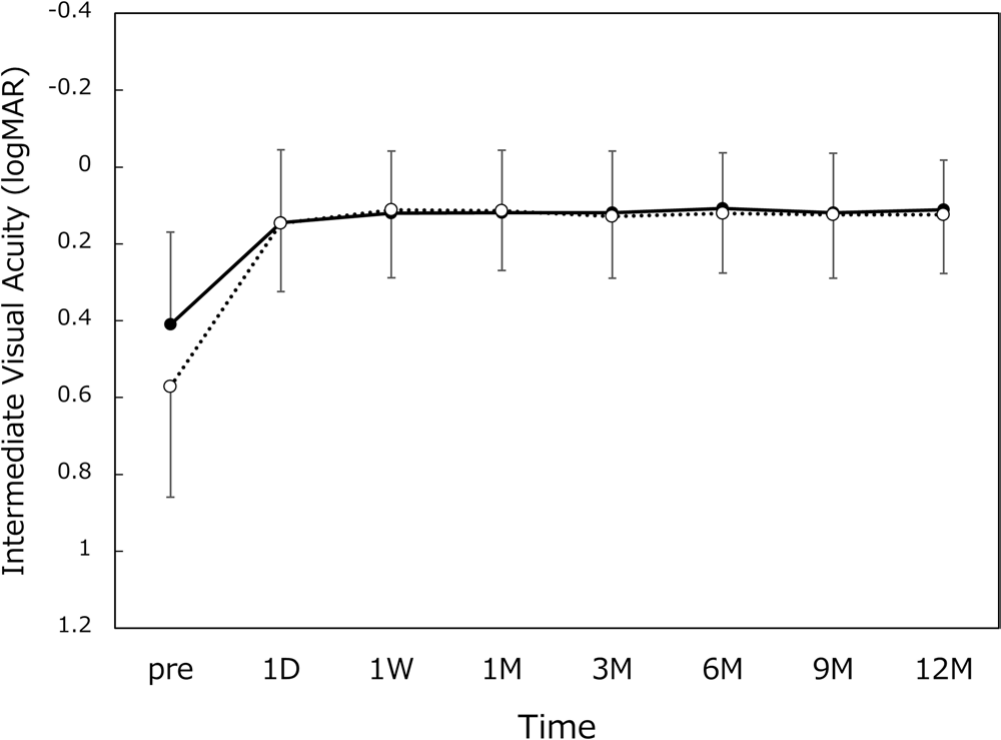
Uncorrected (UIVA, dotted line) and distance-corrected (DCIVA, solid line) intermediate visual acuity. logMAR = logarithm of minimum angle of resolution, mean ± standard deviation.

**Figure 3:**
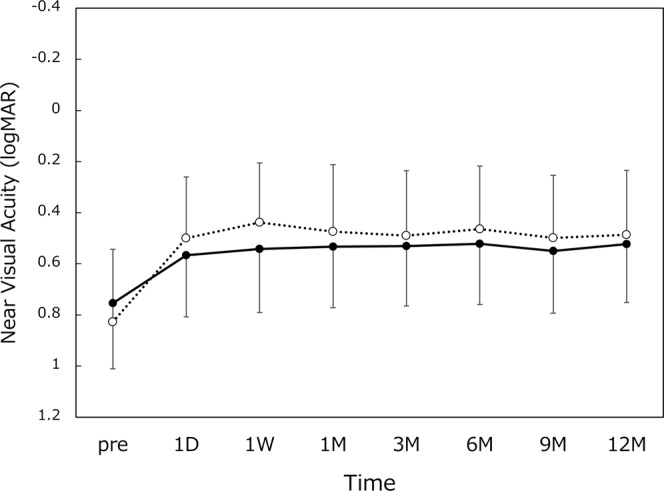
Uncorrected (UNVA, dotted line) and distance-corrected (DCNVA, solid line) near visual acuity. logMAR = logarithm of minimum angle of resolution, mean ± standard deviation.

All 65 patients (120 eyes) completed 12-month follow-up examinations. Preoperatively, axial length was 23.45 ± 0.99 (range 21.47~27.11) mm and radius of curvature of the cornea was 7.65 ± 0.25 (range 7.10~8.23) mm. The time course of changes in distance visual acuity is shown in Fig. 1. Postoperative distance visual acuity was very good throughout 12 months, with UDVA and CDVA of around 20/20 and 20/16, respectively. The measurement results of intermediate visual acuity are presented in Fig. 2. UIVA and DCIVA of approximately 20/25 were attained after surgery. The level of near visual acuity was lower compared with distance and intermediate visual acuity (Fig. 3). UNVA and DCNVA remained at approximately 20/60 and 20/70, respectively.

**Figure 4:**
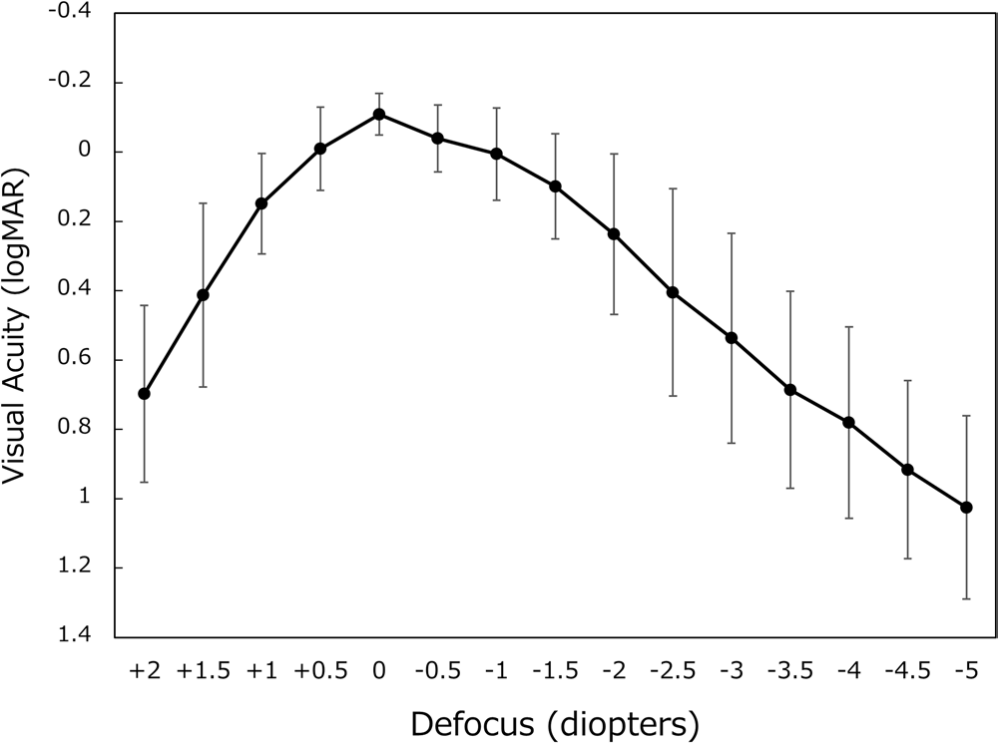
Defocus curve between a spectrum of +2.0 and −5.0 diopters. logMAR = logarithm of minimum angle of resolution, mean ± standard deviation.

The defocus curve measured at 6 months postoperatively is shown in Fig. 4. Visual acuity level of 20/25, 20/30, and 20/40 was attained in the defocus range of +0.5 to −1.5 D, +1.0 to −1.5 D, and +1.0 to −2.0 D, respectively. These results indicate that postoperative uncorrected visual acuity of 20/25 and 20/40 were obtained at as close as 67 cm and 50 cm in distance, respectively.

**Figure 5:**
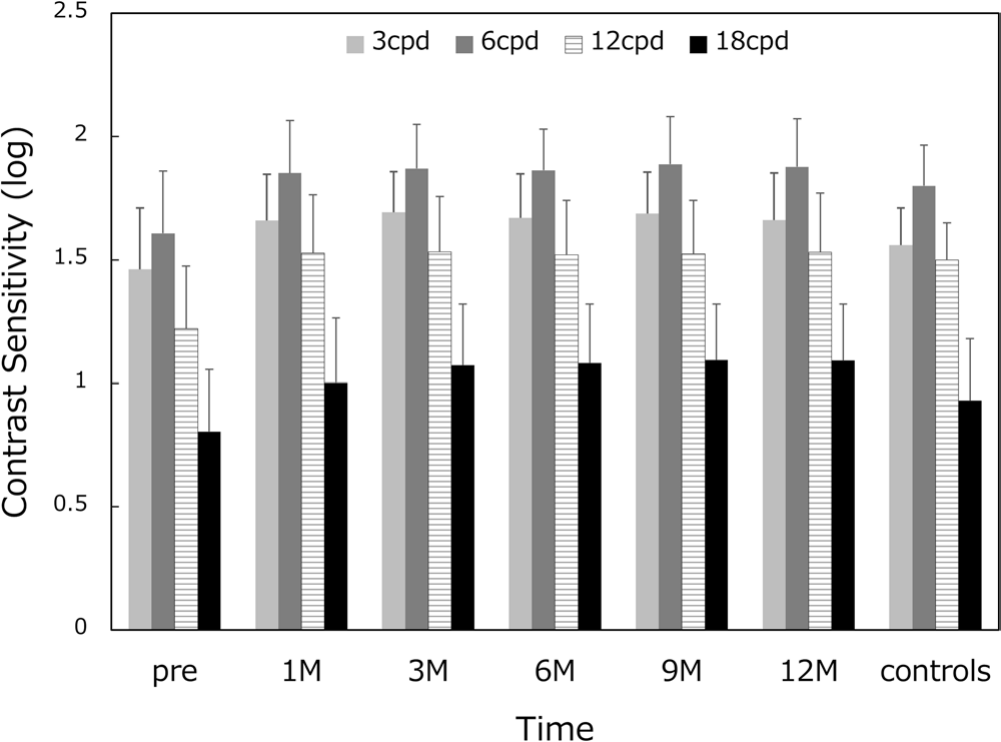
Mean contrast sensitivity function. On each occasion, contrast sensitivity was measured at four spatial frequencies; 3, 6, 12, and 18 cycles per degree (cpd).

Contrast sensitivity function was measured before and 1, 3, 6, 9, 12 months after surgery, and compared with that of age-matched normal controls published on the manufacturer’s web page (Population Norms, Older Age Group: 50–75 Years of Age, http://www.vectorvision.com/csv1000-norms/). All postoperative values were within the normal range (Fig. 5).

**Figure 6:**
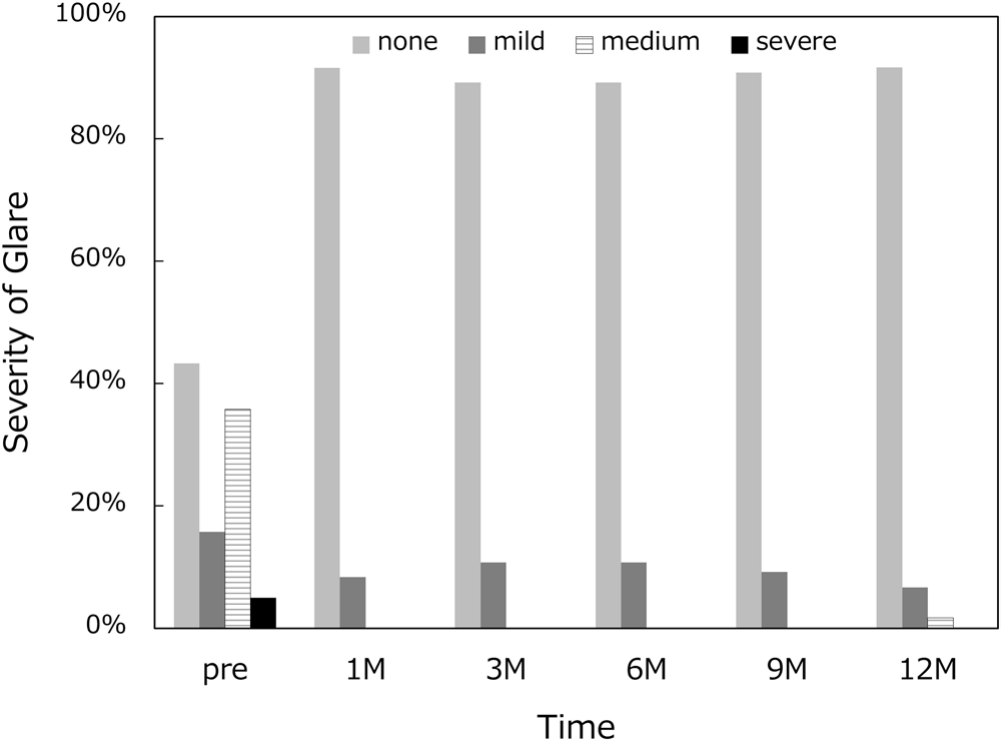
Incidence and severity of glare.

**Figure 7:**
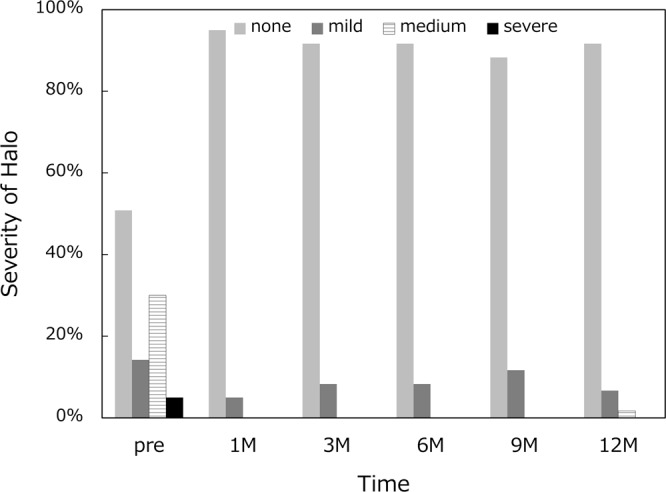
Incidence and severity of halo.

**Figure 8:**
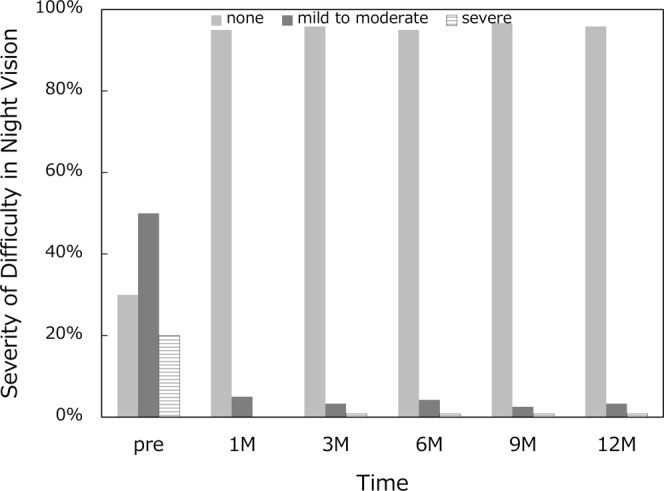
Incidence and severity of difficulty in night vision.

**Figure 9:**
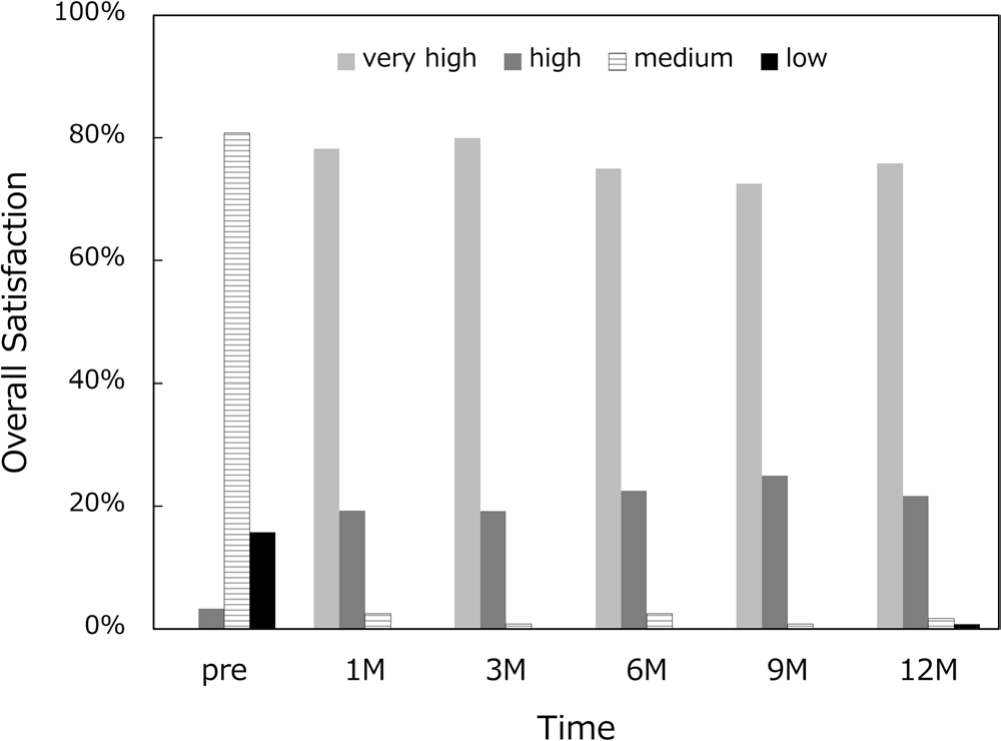
Patient’s subjective satisfaction.

Frequency and severity of photic phenomena are summarized for glare (Fig. 6), halo (Fig. 7), and difficulty in night vision (Fig. 8). There were no severe subjective disturbing symptoms. When asked about the level of overall satisfaction, most of the patients answered very high or high (Fig. 9).

The average corneal endothelial cell density before and 6, 9, and 12 months after surgery was 2,587 ± 291/mm^2^, 2,524 ± 300 /mm^2^, 2,555 ± 319/mm^2^, and 2,532 ± 321/mm^2^, respectively. There was no intraoperative complication. Posterior capsule opacification developed in 9 eyes (7.5%), among which one eye (0.8%) underwent laser posterior capsulotomy. No other significant postoperative complications were reported.

## Discussion

In this study, we followed up 120 eyes for 12 months and examined the time course of changes in visual function. The distance visual acuity was maintained at a very high level throughout the 1-year study period; UDVA and CDVA were around 20/20 and 20/16, respectively. The intermediate visual acuity was also highly satisfactory; both UIVA and DCIVA remained at approximately 20/25. On the other hand, near visual acuity ended up at a lower level compared with distance and intermediate visual acuity. UNVA and DCNVA were approximately 20/60 and 20/70, respectively. This level of near visual acuity is enough to read large print sizes, yet it is not possible to read regular small prints, resulting in a need for reading aids. Kretz FT *et al*. reported that asymmetric +1.5 D near addition multifocal IOLs offered good results for distance visual function (mean UDVA of 0.00 logMAR and CDVA of -0.08 logMAR) in combination with good performance for intermediate distance (UIVA at 80 cm of 0.01 logMAR) and functional results for near distance (UNVA at 40 cm of 0.41 logMAR)^6^. Pedrotti E *et al*. showed that Lentis Comfort LS-313 MF15 provided good distance and intermediate vision (UDVA of −0.01 logMAR and UIVA at 70 cm of 0.05 logMAR), while near visual acuity remained relatively low (UNVA at 30 cm of 0.54 logMAR)^8^. Our results are consistent with the findings of these previous studies.

We measured a defocus curve at 6 months postoperatively. As shown in Fig. 4, the curve showed a gradual decrease from distance to near, unlike a 2-peak curve observed with conventional distance/near bifocal IOLs^5^. The obtained curve demonstrates that uncorrected visual acuity of 20/25 or better was attained at as close as 67 cm (−1.5 D), and 20/40 or better at 45 cm (−2.2 D) in distance. Previous studies also described similar single peak defocus curve associated with Lentis Comfort LS-313 MF15 IOL^6–9^. Aliό JL *et al*. reported consistent results with a different model of rotationally asymmetry multifocal IOL (Lentis-Mplus LS-312 MF15), indicating that patients with this IOL were able to compensate for different levels of defocus and thus obtain good vision at different distances^13,14^. Defocus levels of 0.0 D, −1.5 D, and −3.0 D would correspond to distance, intermediate, and near vision, respectively. Thus, the defocus curve obtained herein well illustrates the design concept of this IOL, meeting the criteria of enhanced depth of focus IOLs.

Contrast sensitivity function measured after surgery was within the range of age-matched normal controls throughout the 1-year study period (Fig. 5). Previous studies reported that this IOL showed contrast sensitivity similar to that of monofocal IOLs^8,9^. It has been demonstrated that contrast sensitivity was not different between extended-range-of-vision IOLs and monofocal IOLs, but these two IOLs showed significantly better contrast sensitivity than multifocal IOLs with +2.0 D and +3.0 D addition^15^. Contrast sensitivity measures a person’s ability to detect low contrast images, unlike the visual acuity test which measures how well a person can identify high contrast black-on-white letters. Using these low contrast images, contrast sensitivity can evaluate subtle changes in vision that cannot be revealed by the visual acuity test. It is a well-recognized subjective parameter for the assessment of quality of vision in patients implanted with premium IOLs.

Frequency and severity of photic phenomena were assessed, including glare, halo, and difficulty in night vision. There were no severe subjective disturbing symptoms, and mild photic phenomena were reported only by a small percentage of patients. Previous studies also reported that few patients complained of disturbing photic phenomena after implantation of this segmented, rotationally asymmetric multifocal IOL with +1.5 D addition^5,7,8^. Yoo A *et al*. exhibited that this refractive multifocal IOL with +1.5 D near addition showed significantly fewer glare and halo symptoms than the similar style of multifocal IOL with +3.0 D near addition^5^. In patients implanted with conventional multifocal and trifocal IOLs, it has been shown that difficulties associated with photic phenomena decreased significantly over time^16,17^. In the present study, there was no fluctuation over time in the severity and incidence of photic phenomena. The level of satisfaction hovered at a high level throughout the 1-year study period. In our patients, disturbing subjective symptoms were infrequent in the first place, and thus there was no need of neuroadaptation for patients to overcome those symptoms. The low-add design of this IOL allows for an elongated focal area without multiple foci as shown by the defocus curve, which minimizes the distinct out-of-focus images generating halos. This may explain the low incidence of disturbing photic phenomena associated with this IOL.

The current study was limited by the lack of a control group. Ideally, it is better to conduct head-to-head comparison with other multifocal or monofocal IOLs. The current study design was agreed on with and approved by the Ministry of Health, Labour and Welfare of Japan, as an open-label, comparative study with past studies on other IOLs.

In conclusion, the current prospective, multicenter study indicated that rotationally asymmetrical multifocal IOL with +1.5 D near addition provides excellent distance and intermediate visual acuity, with high contrast sensitivity and a low amount of subjective photic phenomena. Near visual acuity was not enough for reading small prints, resulting in a need for reading aids. These results are consistent with the theoretical optical properties of this IOL. These findings were stable throughout the 1-year study period.

## Supplementary information

Trial protocol

## Data Availability

The datasets generated during and/or analysed during the current study are not publicly available due to the filing process of this products for approval from the Ministry of Health, Labour and Welfare of Japan, but are available from the corresponding author on reasonable request.
