# Supplementary material for: One-year clinical evaluation of rotationally asymmetric multifocal intraocular lens with +1.5 diopters near addition
Source: Sci Rep. 2019 Sep 11;9:13117. doi: 10.1038/s41598-019-49524-z (PMC6739307; doi:10.1038/s41598-019-49524-z)
Supplement: Supplementary file 1 — Trial protocol [file 41598_2019_49524_MOESM1_ESM.docx]

**Trial Protocol**

**Project summary**

The aim of this study is to assess clinical outcomes after implantation of segmented, rotationally asymmetric multifocal intraocular lens (IOL), Lentis Comfort LS-313 MF15 (Oculentis GmbH, Berlin, Germany). This IOL is a plate-haptic, rotationally asymmetric, refractive multifocal IOL, combining an aspheric distance vision zone and a sector-shaped near vision zone with an add power of +1.5 D on the lens plane. This is a prospective multicenter phase III clinical trial of 12-month duration to file for approval from the Ministry of Health, Labour and Welfare of Japan. Patients eligible for cataract surgery and IOL implantation at six surgical centers are recruited for the study. The ophthalmological examinations are performed before and 1 day, 1 week, 1, 3, 6, 9, and 12 months after surgery. Uncorrected (UDVA) and corrected (CDVA) distance visual acuity, uncorrected (UIVA) and distance-corrected (DCIVA) intermediate visual acuity at 70 cm, and uncorrected (UNVA) and distance-corrected (DCNVA) near visual acuity at 30 cm are measured. A defocus curve is obtained and patients are asked about the severity of photic phenomena. Expected outcomes are good distant and intermediate visual acuity, low incidences of photic phenomena, and high level of patient satisfaction.

**General information**

**Protocol title, protocol identifying number, and date**

ClinicalTrials.gov. A Study Assessing Safety and Efficacy of MD-15 Intraocular Lens in Patients With Aphakic Eye After Cataract Surgery (https://clinicaltrials.gov/ct2/home); Identifier: NCT02888210.

Available at: https://clinicaltrials.gov/show/NCT02888210 (September 2, 2016).

**Name and address of the sponsor/funder**

Santen Pharmaceutical. Grand Front Osaka Tower A, 4-20 Ofuka-cho, Kita-ku, Osaka 530-8552, Japan.

**Name and title of the investigator(s) who is (are) responsible for conducting the research, and the address and telephone number(s) of the research site(s), including responsibilities of each**

Hiroyuki Arai, MD. Queen’s Eye Clinic, 2-3-5 Minatomirai, Nishi-ku, Yokohama, Kanagawa 220-6204, Japan, Tel +81-45-682-4455. Responsibility: performance of surgery, data collection, and approval of final manuscript.

Yoshifumi Fujita, MD. Fujita Eye Clinic, 6-27 Sakorokubancho, Tokushima, Tokushima, 770-0026, Japan. Tel +81-800-222-1010. Responsibility: performance of surgery, data collection, and approval of final manuscript.

Mikio Inamura, MD. Inamura Eye Clinic, 5-125-2F, Isezakicho, Naka-ku, Yokohama, Kanagawa, 231-0045, Japan. Tel +81-45-263-1771. Responsibility: performance of surgery, data collection, and approval of final manuscript.

Yasushi Inoue, MD. Inoue Eye Clinic, 1-14-31 Uno, Tamano, Okayama, 706-0011, Japan. Tel +81-863-31-1030. Responsibility: performance of surgery, data collection, and approval of final manuscript.

Toru Noda, MD. Department of Ophthalmology, National Hospital Organization, Tokyo Medical Center, 2-5-1, Higashigaoka, Meguro-ku, Tokyo, 152-8902, Japan. Tel +81-3-3411-0111. Responsibility: performance of surgery, data collection, and approval of final manuscript.

Kazunori Miyata, MD. Miyata Eye Hospital, 6-3 Kuraharacho, Miyakonojo, Miyazaki, 885-0051, Japan. Tel +81-986-22-1441. Responsibility: performance of surgery, data collection, and approval of final manuscript.

**Name(s) and address(es) of the clinical laboratory(ies) and other medical and/or technical department(s) and/or institutions involved in the research**

Tetsuro Oshika, MD, Department of Ophthalmology, Faculty of Medicine, University of Tsukuba, 1-1-1 Tennoudai, Tsukuba, Ibaraki, 305-8575 Japan, Tel +81-29-853-3148. Responsibility: designing of study protocol, data analysis, and preparation of manuscript.

**Rationale & background information**

Conventional multifocal IOLs with two distinct foci can provide patients with good distance and near vision, but intermediate vision is not satisfactory.^1-4^ These IOLs share the disadvantage of offering sharp vision only within a limited zone around the foci. In addition, the main drawbacks of conventional multifocal IOLs is the incidence and entity of reported side effects, including dysphotopsia and loss of contrast sensitivity.^1-4^ There has been a growing demand in intermediate distance activities in everyday life, especially due to the more frequent use of personal computers and smartphones, leading to increased desire for spectacle independence in the intermediate range.

One trend that is surfacing today is the use of low-addition multifocal IOLs to boost intermediate performance and provide patients with good contrast sensitivity. One such IOL is the Lentis Comfort LS-313 MF15, a rotationally asymmetric multifocal IOL with +1.5 diopters (D) near addition. It is a plate-haptic IOL with a refractive segmented multifocal optic. Its concept is to provide patients with enhanced vision at a distance of 60 cm and more. There have been only a few studies which evaluated the clinical performance of this IOL.^5-9^ In general, studies reported good visual performance in distance and intermediate distances, in combination with low incidence of subjective disturbing photic phenomena. In these studies, however, the number of eyes evaluated were small, 21 to 60 eyes, and follow-up period was short, 3 to 6 months, except for one study which assessed 42 patients for 12 months.^8^ We conduct the current prospective, multicenter study to assess the clinical performance of plate-haptic, rotationally asymmetric multifocal IOLs with +1.5 D near addition in a larger cohort for 1 year.

**References (of literature cited in preceding sections):**

1. de Vries NE, Nuijts RM. Multifocal intraocular lenses in cataract surgery: literature review of benefits and side effects. J Cataract Refract Surg 39, 268-278 (2013).

2. Braga-Mele R, Chang D, Dewey S, Foster G, Henderson BA, Hill W, Hoffman R, Little B, Mamalis N, Oetting T, Serafano D, Talley-Rostov A, Vasavada A, Yoo S; ASCRS Cataract Clinical Committee. Multifocal intraocular lenses: relative indications and contraindications for implantation. J Cataract Refract Surg 40, 313-322 (2014).

3. Charman WN. Developments in the correction of presbyopia II: surgical approaches. Ophthalmic Physiol Opt 34, 397-426 (2014).

4. Alio JL, Plaza-Puche AB, Férnandez-Buenaga R, Pikkel J, Maldonado M. Multifocal intraocular lenses: An overview. Surv Ophthalmol 62, 611-634 (2017).

5. Yoo A, Kwag JY, Song IS, Kim MJ, Jeong H, Kim JY, Tchah H. Comparison of visual function after implantation of inferior sector-shaped intraocular lenses: low-add +1.5 D vs +3.0 D. Eur J Ophthalmol 26, 607-611 (2016).

6. Kretz FT, Khoramnia R, Attia MS, Koss MJ, Linz K, Auffarth GU. Clinical evaluation of functional vision of +1.5 diopters near addition, aspheric, rotational asymmetric multifocal intraocular lens. Korean J Ophthalmol 30, 382-389 (2016).

7. Vounotrypidis E, Diener R, Wertheimer C, Kreutzer T, Wolf A, Priglinger S, Mayer WJ. Bifocal nondiffractive intraocular lens for enhanced depth of focus in correcting presbyopia: Clinical evaluation. J Cataract Refract Surg 43, 627-632 (2017).

8. Pedrotti E, Mastropasqua R, Bonetto J, Demasi C, Aiello F, Nucci C, Mariotti C, Marchini G. Quality of vision, patient satisfaction and long-term visual function after bilateral implantation of a low addition multifocal intraocular lens. Int Ophthalmol 38, 1709-1716 (2018).

9. Kim KH, Kim WS. Visual outcome and patient satisfaction of low-power-added multifocal intraocular lens. Eye Contact Lens 44, 60-67 (2018).

**Study goals and objectives:** To assess the clinical performance of plate-haptic, rotationally asymmetric multifocal IOLs with +1.5 D near addition in a larger cohort for 1 year, by analyzing distance, intermediate, and near vision, incidence of photic phenomena, and patient satisfaction.

**Study Design**

This is a prospective, interventional, single-arm study involving patients underusing surgical treatment of cataract at 6 surgical sites. They are selected from consecutive cases among the clinic population who match our inclusion criteria. Eyes are not included if they had any ocular diseases which can affect surgical outcomes. Eyes having corneal astigmatism greater than 1.5 D are also excluded from the study. The ophthalmological examinations are performed before and 1 day, 1 week, 1, 3, 6, 9, and 12 months after surgery.

**Methodology**

Surgeries are performed using a standard technique of sutureless phacoemulsification through a 2.3- or 2.4-mm incision. Anterior capsulorhexis of approximately 5.0 mm in diameter is created and the IOL is implanted into the capsular bag using a Viscojet-BIO 2.2 injector (Medicel AG, Wolfhalden, Altenrhein) and ACCUJECT UNIFIT WJ-60M II (Santen Pharmaceutical Co., Ltd. Osaka, Japan). All eyes are targeted emmetropia.

Preoperative examination include measurements of uncorrected (UDVA) and corrected (CDVA) distance visual acuity, uncorrected (UIVA) and distance-corrected (DCIVA) intermediate visual acuity measured at 70 cm, uncorrected (UNVA) and distance-corrected (DCNVA) near visual acuity measured at 30 cm, manifest refraction, intraocular pressure, slitlamp anterior segment examination, optical biometry, keratometry, and retina evaluation under pupil dilation.

Postoperatively, UDVA, CDVA, UIVA, DCIVA, UNVA, and DCNVA are evaluated at all postoperative visits. A defocus curve for 15 different levels of defocus from +2.0 to -5.0 D in steps of 0.5 D is recorded at 6 months postoperatively. The contrast sensitivity is assessed at 3, 6, 12, and 18 cycles per degree using a CSV-1000 chart (Vector Vision, Greenville, OH) at 1, 3, 6, 9, 12 months postoperatively. To plot the curve, the measurement results are converted to log units using a specific table for the CSV-1000. Corneal endothelial cell count is assessed before and 6, 9, and 12 months after surgery.

Patients are asked about the severity of photic phenomena. The intensity of glare and halo is graded as none, mild, moderate, or severe. The severity of difficulty in night vision is grated as none, mild to moderate, or severe. Patients are also asked about their overall satisfaction with the outcomes as follows, very high, high, medium, and low. The occurrence of any intraoperative and postoperative complications areere recorded throughout the study period.

**References (of literature cited in preceding sections):**

**Safety Considerations**

Any complications during surgery and adverse effects after surgery throughout the follow-up period are carefully monitored and kept in records.

**Follow-Up**

The ophthalmological examinations are performed before and 1 day, 1 week, 1, 3, 6, 9, and 12 months after surgery.

**Data Management and Statistical Analysis**

The patients’ personal information is masked at each surgical sites is handed to University of Tsukuba where statistical analysis is performed. Judging from the previous report that UIVA at 80 cm in eyes with Lentis-Mplus LS-312 MF15 IOL was 0.19 ± 0.11 logMAR, the mean DCIVA at 70 cm in this study was estimated to be 0.17 logMAR. In order that the lower endpoint of 95% confidence interval of DCIVA in our study significantly exceeds the mean DCIVA in eyes with conventional monofocal IOL (0.22 logMAR), the required number of cases was calculated to be 77 eyes (α=0.05, power 80%). In addition, ISO 11979-7:2014 (E) Annex B indicated that 100 eyes are required to statistically compare the rate of eyes with CDVA of 20/40 (0.3 logMAR) or better with the SPE rate (92.5%). A dropout rate of 10% was anticipated, giving the target sample size of 110 eyes.

**Quality Assurance**

The institutional review boards at all sites approved the study protocol, and a written informed consent is obtained from each patient before the study started. The study adhere to the tenets of the Declaration of Helsinki and good clinical practice for a medical device in Japan (Pharmaceuticals and Medical Devices Agency: PMDA clinical trial identifier: TC2). This study is registered at ClinicalTrials.gov ID: NCT02888210, https://clinicaltrials.gov/show/NCT02888210 (September 2, 2016).

**Expected Outcomes of the Study**

This study is aimed at confirming the effectiveness and safety of this IOL. Good distance and intermediate vision, low incidence of photic phenomenon, and high patient satisfaction are expected. By proving this hypothesis, this study will contribute to the advancement of practice in cataract and IOL surgery.

**Dissemination of Results and Publication Policy**

The results will be submitted to peer-reviewed journal with Dr. Oshika as the first author. The data will also be presented at the Annual Meeting of Japanese Ophthalmological Society.

**Duration of the Project**

The surgery was performed between September 2016 to December 2017, and the follow up study was done for 1 year surgery.

**Problems Anticipated**

**Project Management**

Dr. Oshika is responsible for designing study protocol, data analysis, and preparation of manuscript.

Drs. Arai, Fujita, Inamura, Inoue, Noda, and Miyata are responsible for planning of the study, performance of surgery, data collection, and approval of final manuscript.

**Ethics**

The study protocol was reviewed and approved by the Institutional Review Board of each surgery site. This study is conducted in accordance with the Declaration of Helsinki. An informed consent is obtained from all patients in a written form.

**Informed Consent Forms**

The informed consent forms both in English and the local language are attached.
